# Holding collaboration together: a community response using a violence threat and suicide prevention protocol

**DOI:** 10.3389/fpubh.2026.1933435

**Published:** 2026-09-16

**Authors:** Sharon Friesen, Jacqueline Yu, Stephen MacGregor

**Affiliations:** Werklund School of Education, University of Calgary, Calgary, AB, Canada

**Keywords:** collective impact, coordinated community response, interagency collaboration, multi-sector collaboration, violence trauma and suicide prevention protocol, youth mental health

## Abstract

Coordinated, multi-sectoral community responses to youth mental health represent a promising yet undertheorized approach to addressing the service fragmentation that characterizes collaborative work across education, health, policing, and community services. This article presents qualitative findings from a larger sequential mixed-methods study of Strathcona County’s Violence, Trauma, and Suicide Prevention (VTSP) Protocol. The VTSP Protocol is a coordinated community framework designed to address youth mental health, community safety, and wellbeing in Alberta, Canada. The larger study combined social network analysis with qualitative inquiry; this article reports on the qualitative strand, which comprised 12 semi-structured interviews with key stakeholders purposively sampled from across the VTSP network. Thematic analysis of interview transcripts identified four interwoven themes that together describe the relational, communicative, and capacity-building labours through which the protocol is sustained: leadership as relational infrastructure, shared processes and communication structures, cross-sectoral relationships and joint purpose, and capacity building and shared language. Across all four themes, collaboration emerged not as a stable institutional achievement but as a fragile accomplishment, strong yet precarious, requiring continual renewal rather than resting on structural stability. The findings are theoretically grounded in Ecological Systems Theory and Relational Coordination Theory. Implications for the design, sustainability, and transferability of coordinated community response models in other communities and school authorities are discussed.

## 1 Introduction

On February 10, 2026, five students and an educator were killed at Tumbler Ridge Secondary School in northeastern British Columbia in one of the deadliest school shootings in Canadian history, second only to the *École Polytechnique* massacre of 1989 (1, 2). The perpetrator, an 18-year-old who had dropped out of school 4 years earlier, had a documented history of mental health concerns and had been the subject of multiple prior police contacts related to mental health (2, 3). In the days following the tragedy, British Columbia Premier David Eby publicly stated that he would be seeking information from the provincial health system about what interactions it may have had with the perpetrator, a question that laid bare one of the most consequential failures in Canadian community safety: the absence of coordinated, sustained communication among the organizations responsible for identifying and supporting young people at risk (4). Tumbler Ridge is not an isolated case. The 2016 La Loche school shooting in Saskatchewan, in which a 17-year-old with known cognitive and social difficulties killed four people, raised the same fundamental question: what might have been different if schools, health services, police, and community organizations had been working together, sharing information through common protocols, and taking coordinated action before a crisis became a catastrophe (1). These events, rare by international standards but devastating in their impact, are powerful reminders that the systems responsible for youth mental health, community safety, and violence prevention are often fragmented precisely when integration is most urgently needed.

The urgency that these tragedies reveal is inseparable from a broader pattern of deteriorating youth mental health across Canada. Data from the 2023 Canadian Health Survey on Children and Youth indicate that the proportion of young people aged 16 to 21 rating their mental health as fair or poor more than doubled between 2019 and 2023, rising from 12 to 26% (5). Longitudinal surveillance data document sustained increases in diagnosed mood and anxiety disorders, suicidality, and mental health service utilization among youth aged 12 to 24 between 2011 and 2018 (6), and Canadian Institute for Health Information (7) data indicate that mental health disorders account for approximately 5% of all emergency department visits among children and youth aged 5 to 24. The burden is not evenly distributed. According to the 2022 Mental Health and Access to Care Survey, 56% of 2SLGBTQ + youth met diagnostic criteria for a mental health or substance use disorder, and Indigenous youth experience depression and suicide at rates 5 to 7 times higher than those of non-Indigenous peers (8). For these young people, early identification and coordinated support are not merely beneficial; they are potentially lifesaving.

Despite the scale and urgency of this challenge, the systems responsible for addressing youth mental health remain deeply fragmented. Schools, health services, policing, and community organizations each carry distinct mandates, operate under separate legislation and governance structures, and frequently use incompatible professional languages and referral pathways. Surveys of educators consistently identify the lack of coordination between schools and community mental health services as a primary barrier to effective student support (9, 10). Research from Sweden, examining cross-boundary collaboration in youth mental health systems, concluded that there is little scientific evidence on how these collaborations are applied or sustained, and that significant gaps persist between the needs of young people and the quality and accessibility of available services (11). In the Canadian context, these gaps are compounded by workforce shortages, uneven regional resourcing, and the absence of a national framework for coordinating cross-sector responses to youth mental health (10). The result is a system in which information about a young person in crisis rarely travels across the organizational boundaries that divide education from health, health from policing, and policing from community services. The consequences of that fragmentation are not abstract.

What makes coordinated, multi-sector community responses both compelling and rare is precisely the organizational complexity they must overcome. Bringing together education, health, policing, and community services around a shared protocol, common language, and sustained relational infrastructure requires deliberate design, ongoing resourcing, and the cultivation of trust across boundaries that ordinarily separate these sectors. In practice, most communities do not have such systems in place. Where they do exist, they typically involve a small number of organizations operating under limited agreements. The Strathcona County Violence, Trauma, and Suicide Prevention (VTSP) Protocol is an exceptional case: a coordinated community framework in which 16 organizations from across education, health, policing, and community services have worked together, using the Violence Threat Risk Assessment (VTRA) and Threat Evaluation of Situations (TES) processes as shared methodology and language, to address youth mental health, community safety, and wellbeing. Operationally, the protocol is sustained through regular cross-agency meetings, shared processes and forms, designated community leads, and ongoing VTRA and TES training; when coordinated action is required, community leads help gather information, bring relevant partners together, and guide the collaborative process. The protocol was built, in part, in direct response to a traumatic community event, and it has received national recognition as a promising model for other communities and school authorities in Alberta and across Canada. Its existence represents an organizational and relational achievement that the vast majority of Canadian communities have not yet attained.

This article reports qualitative findings from a larger sequential mixed-methods study of the VTSP Protocol. The larger study combined social network analysis of 25 respondents across 16 core organizations with the qualitative inquiry reported here. The qualitative strand comprised 12 semi-structured interviews with key stakeholders drawn from across education, policing, and community services within the VTSP network.

While a growing body of scholarship addresses multi-sector collaboration and collective impact in social and health systems (12–14), three important gaps remain in the literature. First, existing research attends predominantly to the structural conditions that enable collaboration to begin, rather than to the relational, communicative, and capacity-building labour through which it is maintained and renewed over time. How collaboration is sustained across multiple sectors, particularly in communities that must navigate staff turnover, jurisdictional ambiguity, information-sharing legislation, and periodic crises, remains comparatively undertheorized. Second, qualitative research that examines the lived experience of practitioners working within coordinated community protocols that span education, health, policing, and community services is scarce. Most collaborative governance research operates at the level of policy analysis or program evaluation; the practitioner perspectives that illuminate how shared processes, trust, leadership, and language function in daily practice are underrepresented. Third, in-depth, community-specific qualitative research on coordinated community responses to youth mental health in the Canadian context is limited, particularly in jurisdictions governed by Alberta’s Protection and Privacy Act and Access to Information and Privacy, where legislative conditions create distinct dynamics for cross-sector information sharing that may differ meaningfully from those studied in the United States and Europe.

This article addresses these gaps by offering a theoretically grounded, qualitative account of how collaboration is sustained within a coordinated community response to youth mental health and community safety. Drawing on thematic analysis of 12 in-depth interviews, the study identifies four interwoven themes: leadership as relational infrastructure, shared processes and communication structures, cross-sectoral relationships and joint purpose, and capacity building and shared language. A central interpretive finding is that collaboration within the VTSP Protocol is not a stable institutional achievement but a fragile accomplishment, strong yet precarious, requiring continual renewal rather than resting on structural stability alone. In a national context where the consequences of fragmented community responses can be measured in lives, this finding carries direct implications for how coordinated community responses to youth mental health are designed, resourced, and sustained over time.

### 1.1 Guiding questions and system-level relevance

The research questions that guided this study were developed in response to the evidence that coordinated community responses to youth mental health, though demonstrably valuable, are rarely examined in depth from the perspective of those who sustain them. Understanding how collaboration is practiced and maintained within such a protocol, and what strategic and relational conditions underpin its success, has direct implications for communities seeking to build or strengthen similar models. This article is oriented by two research questions:

- How do the key stakeholders and organizations within Strathcona County’s VTSP Protocol collaborate and exchange information to address the mental health and wellbeing of children and youth?
- What are the underlying strategies and relational dynamics that contribute to the success of Strathcona County’s VTSP Protocol as a coordinated community response, and how can these insights inform the development of similar models for other communities and school authorities in Alberta?

These questions are grounded in a recognition that the VTSP Protocol operates as a system-level response to challenges that no single organization can address alone. Youth mental health, violence prevention, and community safety sit at the intersection of education, health, policing, and community services, and effective responses require that these sectors share information, align their actions, and sustain coordination across the organizational boundaries that ordinarily separate them. The first research question attends to the structural and relational dimensions of that coordination, examining how collaboration and information exchange are enacted in practice across 16 organizations with distinct mandates and professional cultures. The second question moves from description to explanation and implication, probing the deeper strategies and relational conditions that enable the protocol to function as a coherent, sustained response rather than an episodic or fragmented one, and asking what lessons can be drawn for the many Alberta communities and school authorities that have not yet developed such capacity.

The system-level relevance of this inquiry extends beyond Strathcona County. At a time when the deterioration of youth mental health is well documented nationally (6, 15), and when high-profile tragedies have repeatedly exposed the costs of fragmented community responses, the question of how to build, sustain, and transfer integrated multi-sector frameworks has acquired considerable urgency. This study contributes to that question by providing a theoretically grounded, qualitative account of the relational, communicative, and capacity-building labours that hold such a framework together, offering both conceptual insight and practical implications for communities, school authorities, and policymakers seeking to strengthen cross-sector coordination in support of children and youth.

## 2 Theoretical framework

This study is grounded in two complementary theories serving distinct purposes: Bronfenbrenner’s Ecological Systems Theory and Relational Coordination Theory. Together, these theories provide a foundation for understanding both why a coordinated community response is necessary and how such a response is sustained in practice. Ecological Systems Theory provides a contextual frame for understanding youth mental health, trauma, violence prevention, and suicide prevention as concerns shaped across multiple interacting environments, while Relational Coordination Theory provides an interpretive lens for examining how organizations working across boundaries coordinate their actions through relationships and communication.

### 2.1 Ecological systems theory

Bronfenbrenner’s Ecological Systems Theory conceptualizes child and youth development as embedded within nested and interacting systems, including the microsystem, mesosystem, exosystem, macrosystem, and chronosystem (16, 17). This theory is useful for the present study because youth mental health, suicide prevention, trauma response, and violence prevention cannot be understood as individual concerns alone. They are shaped through relationships among young people, families, schools, peers, community organizations, health services, policing, and broader social conditions.

The VTSP Protocol reflects an ecological understanding of youth wellbeing. Schools may observe changes in behaviour, attendance, peer relationships, or emotional distress. Police may hold information related to safety concerns or prior incidents. Health and mental health providers may understand clinical or crisis-related needs. Community organizations may know family, cultural, social, or local circumstances that are less visible to formal institutions. Each sector therefore holds partial knowledge, and no single organization can adequately interpret or respond to the whole situation alone.

Ecological Systems Theory helps frame the VTSP Protocol as a response to problems that emerge across systems and therefore require coordinated action across systems. The protocol’s emphasis on education, health, policing, municipal services, and community organizations working together aligns with Bronfenbrenner’s view that development and wellbeing are shaped through interactions among multiple environments. In this study, the ecological lens supports the interpretation of collaboration not as an optional enhancement to service delivery, but as a necessary response to the multi-system nature of youth mental health and community safety.

### 2.2 Relational coordination theory

Relational Coordination Theory provides the primary theoretical lens for understanding how collaboration is sustained within the VTSP Protocol. Developed by Gittell, Relational Coordination Theory explains coordination as a mutually reinforcing process of relating and communicating among people whose work is interdependent (18, 19). The theory identifies three relational dimensions, shared goals, shared knowledge, and mutual respect, and four communication dimensions, frequent, timely, accurate, and problem-solving communication, as central to effective coordination.

Relational Coordination Theory is also useful for examining why interorganizational collaboration may be experienced as both strong and fragile. Coordination is strengthened when interdependent actors develop shared goals and knowledge, mutual respect, and clear pathways for frequent, timely, accurate, and problem-solving communication. Conversely, turnover, uneven participation, unclear responsibilities, inconsistent opportunities to develop shared knowledge, or uncertainty about information sharing can weaken the relational and communicative ties on which coordination depends. The theory therefore provides a useful interpretive lens for understanding both the strength and the potential fragility of coordinated cross-sector work.

The theory is especially useful for interpreting the four themes identified in this study. Leadership as relational infrastructure reflects the work of maintaining shared goals, mutual respect, and problem-solving communication. Shared processes and communication structures reflect the importance of timely, accurate, and frequent communication. Cross-sectoral relationships and joint purpose align with the theory’s emphasis on shared goals and mutual respect. Capacity building and shared language reflect the development of shared knowledge across professional and organizational boundaries.

### 2.3 Integrating the theoretical framework

Together, Ecological Systems Theory and Relational Coordination Theory provide a coherent theoretical framework for this study. Ecological Systems Theory explains why the VTSP Protocol is necessary: youth mental health, trauma, suicide prevention, and violence prevention are shaped across multiple systems and therefore require a multi-system response. Relational Coordination Theory explains how such a response is sustained: through shared goals, shared knowledge, mutual respect, and communication that is frequent, timely, accurate, and oriented toward problem solving.

This theoretical framing also supports the study’s central interpretive finding: collaboration within the VTSP Protocol is not maintained by protocol alone. Formal structures, shared forms, committees, and procedures matter, but they only function when sustained by relationships, communication, trust, and shared understanding. These theories did not provide an *a priori* coding framework for the qualitative analysis. Rather, Ecological Systems Theory provides a conceptual account of the multi-system context in which the VTSP operates, while Relational Coordination Theory is used interpretively to consider the relational and communicative dynamics evident in the inductively developed themes. The VTSP Protocol can therefore be understood as an ecological community response held together through relational coordination. Its strength lies in the capacity of diverse partners to continually renew the relational and communicative conditions required to act together in support of children and youth.

## 3 Literature review

### 3.1 Systemic approaches to public health

Public health approaches provide a common rationale for promotion and prevention efforts focused on youth mental health and community violence (20–22). Public health can be defined as: “An organized activity of society to promote, protect, improve, and when necessary, restore the health of individuals, specified groups, or the entire population” (23). This definition acknowledges a need for systemic coordination to address health promotion, prevention, and intervention efforts, which aligns with multi-tiered service mental health delivery frameworks, informed by Bronfenbrenner’s (16, 17) Ecological Systems theory (22, 24). Ecological Systems theory frames child development through layers of environmental interactions across time, including more direct (e.g., home, school) and indirect influences (e.g., impact of parent’s workplace on home), as well as socio-cultural impacts (17). As such, collaboration across organizations and sectors (e.g., education, healthcare, justice) typically characterizes coordinated efforts to address the complex, systemic factors associated with public health concerns (20, 22, 25).

For community violence and suicide, risk and protective factors cut across these layers of environmental impact (26, 27). For instance, Cuesta et al. (27) conducted an integrative review of factors related to teen bullying and suicide, identifying significant individual (i.e., mental health challenges, substance use, low achievement, prior suicide attempt), household (i.e., family violence, suicide attempt, dysfunction, prior abuse, single parent) and social (e.g., low socio-economic status) variables. In contrast, they found that protective factors included positive mental health, school safety, two-parent households, and positive relationships at home and school (i.e., supportive teacher involvement). The Center for Disease Control and Prevention (CDC) has also mapped the risk and protective factors related to community violence, publishing a community violence prevention resource (20, 26). For community violence, protective factors included the availability of mental health supports, the presence of relational bonds (i.e., family, community, peer, and school), and inter-organizational community-based resource collaboration (26). The CDC (20) recommended seven research-supported strategies to prevent community violence, spanning economic, relational, educational, and intervention-based domains. Moreover, their resource positions multi-sectoral collaboration as a necessity for effective implementation of prevention efforts, identifying a broad list of potential partners (e.g., law enforcement, social services, education systems, religious communities).

### 3.2 Interagency collaboration

School-community partnerships have become a hallmark of several frameworks that call for greater service integration to address the physical, mental and behavioural health needs of youth (24, 28, 29). Interagency collaboration refers to collective efforts across distinct and often multidisciplinary services to achieve a common purpose (30). For instance, the CDC and Association for Supervision and Curriculum Development developed The Whole Child, Whole School, Whole Community framework, which emphasizes coordination between health and education sectors (29). In a systematic review of 33 studies, Cooper et al. (30) identified the most prevalent youth-serving collaborations spanned mental health, school-based, child welfare, and counselling service sectors. However, interagency collaboration has also focused on the prevention of community violence, youth arrests, and suicidality. Related cross-sectoral strategies have included implementing universal socio-emotional programs in schools, primary care screening processes, a police-mental health diversion model, and threat assessment approaches (31–33). Multidisciplinary school teams are common across several school threat assessment models (31). However, Jackson and Viljeon’s (31) review of five different school threat assessment models identified VTRA as one of only two approaches that engaged community-based collaborators alongside school teams. Despite this unique contribution, a dearth of peer-reviewed research has explored the implementation outcomes of VTRA, similar to most threat assessment models included in their review.

Given this dearth of implementation evidence, prior reviews of the literature have explored the factors that foster and hinder inter-organizational collaboration for physical and mental health (30, 34, 35). Related to coordination of services for children’s mental health, prevalent overlapping facilitators of interagency collaboration included inter-organizational trust and shared training or networking opportunities (30, 34). In a systematic review, Cooper et al. (30) found that quality and frequency of communication were one of the leading facilitators of interagency collaboration, whereas challenges in this domain were identified as the second greatest hindrance. In public health partnerships, Wiggins et al. (35) also found communication and information sharing to be frequently reported in studies, suggesting that partners may consider these factors significant. Systematic reviews of the interagency collaboration in health and mental healthcare both identified insufficient resources or funding as a top impediment (30, 35). Across the factors that shape interagency collaboration, Wiggins et al. (35) asserted that overlapping points suggest a relational undercurrent to high quality co-working dynamics, warranting further exploration through this qualitative inquiry.

Related scholarship on collective impact identifies a common agenda, mutually reinforcing activities, continuous communication, shared measurement, and backbone support as conditions that can enable cross-sector collaboration (12). More recent work has emphasized the adaptive, relational, and equity dimensions of collective impact (13, 14). In this study, this literature provides a point of comparison for the findings rather than an analytic framework.

## 4 Materials and methods

### 4.1 Research approach

The overarching study, from which these qualitative findings were drawn, employed a descriptive sequential mixed-methods case study (36). A descriptive sequential mixed-methods design was appropriate because the larger study sought to understand a complex, real-world community response by first mapping patterns of collaboration across the VTSP network and then using qualitative inquiry to examine how those patterns were experienced and sustained in practice (37, 38). The case study orientation was also appropriate because the VTSP Protocol represents a bounded, community-based response involving multiple organizations working across education, health, policing, municipal, and community service sectors.

The larger study proceeded in two phases. In phase one, a social network analysis (SNA) was conducted, drawing on survey results from 25 respondents across 16 core organizations to map out collaborative patterns, trust, organizational learning, and leadership within the VTSP network. This quantitative phase identified key connectors, brokers, and structural features of the collaborative system. In phase two, 12 semi-structured interviews were conducted with key stakeholders identified through the SNA, selected based on measures of brokerage. These interviews explored how leadership, communication, relationships, and capacity building were experienced in practice of Strathcona County’s VTSP Protocol. This article distinctly reports on the qualitative strand of this inquiry to adequately illuminate participants’ viewpoints of their experiences, highlighting the relational dynamics that underpin interagency collaboration.

The interview phase aimed to build on insights generated through earlier stages of the research and was designed to interrogate emergent patterns and dynamics through the lived experiences of key VTSP participants. By focusing on interviewees with strategic positions within the network, the study sought to deepen understanding of how information flows, trust develops, and leadership is enacted across a complex, interagency system addressing child and youth mental health. The interviews were conducted with key informants involved in the VTSP initiative, focusing on those identified as potential knowledge brokers based on their centrality scores in the SNA component of the study (39, 40). Semi-structured interviews were selected for their ability to elicit rich, context-specific insights while maintaining a degree of consistency across participants (41, 42).

### 4.2 Participant recruitment and sample

Through the phase one SNA, 14 respondents were identified as key stakeholders, and all of them had assented in the survey to be contacted for a follow up interview. These prospective interviewees received an email invitation, resulting in 12 total participants recruited in phase two. All 12 interviewees signed an informed consent form, which outlined the purpose of the study, confidentiality procedures, and their rights as participants. These participants represented a range of sectors including education (*n* = 3), policing (*n* = 4), and community services (*n* = 5). This sampling strategy enabled triangulation across multiple organizational vantage points and ensured that the interview data would reflect the complexity of the VTSP Protocol as a coordinated response to child and youth mental health and wellbeing. In line with the Tri-Council Policy Statement: Ethical Conduct for Research Involving Humans (TCPS), this study received approval from the Conjoint Faculties Research Ethics Board (CFREB), University of Calgary (REB24-0457).

### 4.3 Data collection and analysis

Each semi-structured interview was conducted by one of two Principal Investigators, audio-recorded through institutional accounts on the Zoom video conferencing platform. Field notes were taken throughout to supplement the transcripts with contextual observations and were used as contextual aids during interpretation rather than coded as a separate data source. The length of interviews spanned from around 23 to 67 min, resulting in an average interview time of 44 min. The interview guide was organized into three thematic sections (Supplementary Appendix 1):

- *Role and Involvement in the VTSP Protocol*—Participants were asked about their current roles, professional backgrounds, and specific contributions to the design and implementation of the VTSP Protocol.
- *Reflections on Social Network Analysis Findings*—Before this section, participants were provided with a high-level summary of the SNA findings and invited to reflect on their resonance, surprises, and implications for collaboration, trust, leadership, and organizational learning.
- *Emergent and Partner-Generated Questions*—The final section explored experiences with key components of the protocol, such as the Traumatic Event System (TES) model, the perceived durability of the initiative amidst leadership changes, and how participants navigated the complexity of the multi-agency collaboration.

After de-identification, a CFREB approved transcription service, Otter.ai, generated preliminary transcripts of the interviews. A research assistant manually verified the accuracy of the 12 transcripts and uploaded the files into a qualitative data analysis software, NVivo. The first two authors conducted a thematic analysis of the data, drawing on Saldaña’s (43) codes-to-theory model, outlined as follows. That is, data were first coded using three methods: (1) *descriptive codes* to track key topics; (2) *in vivo codes* to retain participants’ verbatim language; and (3) *concept codes* to synthesize key ideas. In the second cycle, *focused coding* consolidated first-round codes into over-arching categories, informing the interpretation of themes across the dataset. Coding was inductive and data-led; the theoretical frameworks were not used to generate predetermined coding categories. Following the development of the themes, Relational Coordination Theory was brought into the interpretive phase to examine how the emergent findings illuminated the relational and communicative conditions through which cross-sector coordination was sustained.

Triangulation and member checking were employed to enhance the validity of our findings (43, 44). Participants across multiple cross-sectoral organizations provided diverse organizational viewpoints, supporting data-source triangulation and data validation (44). As well, theory and investigator triangulation brought multiple theoretical and researcher lenses to the interpretation and memoing of the emergent categories and themes; ongoing dialogue, centered on supporting evidence from the interview transcripts, contributed nuance and consistency to researchers’ joint interpretations (43, 44). Finally, member checking was conducted to verify the alignment between interpretations and participants’ views of their experiences (43).

## 5 Results

The findings from this study are organized into four interwoven themes that together describe how the VTSP protocol was sustained across organizations. While participants spoke of leadership, processes, relationships, and capacity in distinct ways, these dimensions were not separable; each was experienced as fragile and renewable, continually requiring attention to hold collaboration together. The themes that follow trace how participants narrated leadership as relational infrastructure (Theme 1), communication as both enabling and precarious (Theme 2), relationships and joint purpose as the substratum of collaboration (Theme 3), and capacity building and shared language as the means of sustaining alignment (Theme 4). Across these accounts, collaboration emerged as a fragile accomplishment, precarious and dynamic, requiring continual renewal.

Table 1 provides a summary of the four themes and their key messages for quick reference.

**Table 1:** Summary of findings and key messages.

Theme	Key message	Key features
1. Leadership as relational infrastructure	Leadership sustained collaboration through the relational work of holding people and organizations together, while also enabling action during periods of uncertainty.	Backbone and broker roles; convening and connecting partners; sustaining trust; catalytic action when systems or processes were insufficient.
2. Shared processes and communication structures	Shared structures created predictable opportunities for communication and information exchange, but their effectiveness depended on continued participation and use.	VTRA and TES processes; regular meetings and committees; common forms; reachable contacts; challenges related to uneven engagement, information sharing, and follow-up responsibilities.
3. Cross-sectoral relationships and joint purpose	Collaboration rested on pre-existing relationships and a shared commitment to the safety and wellbeing of children and youth; the protocol built upon rather than created this relational foundation.	Relational trust; familiarity across organizations; shared purpose; commitment extending beyond individual organizational interests.
4. Capacity building and shared language	Ongoing training and shared language helped organizations maintain alignment across differences in professional roles, organizational cultures, and personnel changes.	Training and re-education; VTRA and TES language; trauma-informed approaches; onboarding and refreshers; transfer of knowledge across turnover.

### 5.1 Theme 1: leadership as relational infrastructure

When participants spoke of leadership, they rarely described authority, titles, or organizational charts. Instead, their words carried images of holding, carrying, brokering, and acting. Leadership, in their accounts, was not managerial but deeply human: the invisible labour of sustaining fragile relationships and moving work forward in uncertain terrain. Two interwoven aspects emerged from these stories. At times, leadership was catalytic—improvised, urgent, and visionary action that propelled the work forward when systems faltered. In other instances, it was backbone and broker—the quiet, sustaining infrastructure of trust and relationships that allowed collaboration to endure. These aspects were not separate “types” of leadership but different inflections of the same practice, each necessary, each incomplete without the other.

Participants also located the origins of this leadership in a critical moment: the bombing incident that served as “the crux of the protocol.” Several described how this traumatic event created a readiness for collaboration that might not otherwise have emerged. Robin, remembered by participants as the “founding father” of the protocol, embodied this moment of catalysis. Even those who had not worked with her directly referred to her influence through organizational storytelling, underscoring how visionary leadership and the urgency of crisis together seeded the ground for sustained collaboration.

#### 5.1.1 Backbone and broker

Participants often turned to metaphors of structure and rhythm to describe this sustaining work. Madison and Rowan described leadership roles within collaboration, using the term, “backbone.” Madison offered an explanation: “I think a lot of this role as kind of a backbone type role… bridging and brokering relationships between and amongst community partners, being a bit of a sounding board.” The metaphor suggests invisibility and necessity; a backbone is rarely seen, but without it, the whole body collapses. Leadership, in their description, was not about direction but about holding the relational frame so that others can act within it. Rowan used similar language at the system level, noting “the heavy backbone role that FCS plays and putting the resources that they dedicate to it” particularly in “develop[ing] the capacity of our partners that makes our system stronger.” Kim emphasized coordination over command—keeping people at the table and sustaining trust so the work could move forward. Leadership, in this account, was quiet and even mundane: not instructing but making space, sustaining presence, and protecting trust.

Several participants then described what that “holding” looks like in practice. Rae emphasized regular, scheduled touchpoints, committee meetings and stakeholder gatherings that “continue to bring this work forward,” functioning as moments of “grounding back together.” From school-division vantage point, both Kim and Taylor framed this holding as a connective force. For instance, Kim described this intentional leadership as “pulling people together,” but also keeping the work organized, and helping “fill gaps” through training and materials. When a single agency could not convene during an event, Rae explained that the designated Community Lead could “help…collect [information] and gather the pieces together, and…lead the process and the conversation.” Together, these accounts cast “backbone” work as both structural and relational, the practical choreography of convening and the quieter labour of keeping trust warm between meetings.

Leaders shaped the conditions to sustain collaboration. Rae contributed to this understanding by giving leadership a temporal shape. She explained how the 90-day sprint model provided a *cadence of trust* that reassured partners the work would continue: *“*So we will try things for 90 days, and then we will fix it if it needs to be fixed and try it for another 90 days. But at any point in those 90 day cycles, we are feeling that it’s not worth the value and effort that we are putting into it, then we either have to remodel this or...or learn what we can from that experience and put it aside.” Trust, in this account, was not static but rhythmic, kept alive through cycles of return. Alex similarly pointed to the “behind-the-scenes” work in programs and roles that feed the protocol, revealing the inward work that sustains the outward cadence of meetings. These accounts depict leadership as relational infrastructure that allowed collaboration to endure. In effect, leaders were both backbone and keeper of the cadence, holding relationships while pacing cycles of meeting, review, and recommitment.

At the same time, participants cautioned that backbone leadership can become a vulnerability when it depends too heavily on a single coordinator, champion, or agency. Pat warned that collaborative frameworks should not be “overly personally dependent,” because the work must continue when particular people leave or roles change. Morgan translated this risk into practical terms, emphasizing the need for a central contact, approved communication templates, and trained communicators who could prevent bottlenecks during activation. Taylor similarly stressed the importance of documentation and routine outreach so that relationships and processes do not reside with one individual. While participants affirmed FCS’s neutrality as a valuable coordinating anchor, they also argued that the backbone function needed to be more deliberately distributed through shared documentation, codified hand-offs, and rotating or supported leadership roles. Rather than remove backbone leadership, participants suggested that system-level processes should be strengthened as a protective buffer.

#### 5.1.2 Catalytic action

Yet alongside this quiet infrastructure, participants described the need for dependable leaders who could steadily navigate crises with a bold commitment to the community vision. Within the protocol, Taylor described the importance of “choosing your people carefully, who you put in those lead positions.” For Taylor, frontline leadership is governed by “the most responsible person...calm,” capably steadfast amid “high crisis situations that are very unsettling.” Referred to as a “mindset,” Rowan emphasized acting with purpose even amid uncertainty, anchoring actions in safety rather than position. Their words emphasize leadership as an ethical urgency rather than positional legitimacy.

Many participants described the optimal characteristics of leaders. Rowan endorsed leaders who “assume a little bit of risk... confident and humble....not afraid....willing to test the boundaries a little bit.” For Pat, collaboration calls for leadership that is daringly invested in the necessary actions to achieve joint work: “you need leaders that understand the value of collaboration, are prepared to take risks and...to share information for a common, shared goal.” Madison broadened this commitment to a “genuine or authentic belief and investment in not only this work, but...collaboration as a whole.” From her perspective, effective key leaders are skillfully “relational,” but also bold in their “passion for it.... contagious in the best way possible.” Morgan reinforces this catalytic energy in recalling the protocol’s rollout, “which was really exciting, really great. There was a lot of buy-in and a lot of passion for it, and I think that people recognize how important it is. You know, longevity maintenance.” Rae considered this enthusiasm to be the necessary catalyst that sustains momentum: “We need leaders who will champion it, and we need leaders who will keep others motivated, and we need some people who will do the work and not just talk about it.” Alex echoed the productive harmonies between leaders’ investment and frontline efforts: “You need people up at the top to say, we buy in, we believe in this, this matters. And then you need them...to delegate that....where somebody can truly participate in the protocol.” Together, these accounts suggest that catalytic leadership combines ethical urgency with relational commitment: leaders must be willing to act when waiting for formal procedural clarity could increase risk, while also cultivating the shared buy-in needed to sustain the protocol over time. In this view, moving amid uncertainty is less bravado than care, a readiness to move while staying answerable to the shared purpose.

#### 5.1.3 Bringing leadership as relational into view

Taken together, these stories reveal leadership not as positional authority but as relational infrastructure, at once catalytic and sustaining, structural and affective, strong yet fragile. To lead was to be a backbone, bridging and brokering relationships, while also creating a cadence of trust and a visible enthusiasm that kept partners returning, circling back, and staying the course. It was the courage to act anyway when authority was uncertain, the improvisational willingness to keep moving when protocols faltered, and the emotional labour of holding worry, fear, and conflict so others were not left to carry it alone. Participants’ metaphors, including backbone, cadence, acting anyway, and holding, remind us that leadership was less about command than about the fragile labour of sustaining collaboration. They also pointed to the people they called brokers and bridgers: those with big-picture sensibilities who connect the dots, pick up the phone, ask for clarity, and, when needed, challenge respectfully. Madison observed that the genuine belief and investment of such actors can be contagious, facilitating buy-in and helping hesitant partners step in, while Rae emphasized how that energy keeps the work moving. Collaboration, in these accounts, did not rest on managerial control but on the continual renewal of relational infrastructure, sustained through rhythms of trust, courageous improvisation, and the willingness to hold both the work and the people within it. Yet leadership was also described as a point of vulnerability; overreliance on a single coordinator or champion left the system exposed to employee turnover, suggesting that leadership was both the backbone and, at times, the Achilles’ heel of collaboration.

### 5.2 Theme 2: shared processes and communication structures

When participants reflected on how the VTSP protocol held their work together, they spoke of the scaffolds that made information sharing possible: the protocol itself, the use of VTRA and TES processes, the regular meetings and common forms that created a shared language across agencies. These structures were experienced as necessary scaffolds that allowed diverse professionals to return to the table with some measure of continuity. Yet these same stories carried reminders of fragility, FOIP concerns that were not always well understood, uneven engagement that left a few carrying the work, staff turnover that unsettled momentum, and shifting roles that repeatedly demanded re-explanation. What emerged was that collaboration depended on continual renewal: keeping communication alive across protocols, clarifying privacy concerns, and sustaining trust even when the system itself wavered.

#### 5.2.1 Shared structures for trust

Shared structures provided opportunities for communication and alignment among agencies. To establish and maintain collaboration, participants referenced the role of scheduled meetings and instrumental committees that sustained collaboration. Rae pointed to scheduled meetings and an instrumental Stewardship Committee that “continue to bring this work forward,” describing those gatherings as moments of “grounding back together.” Madison connected those routines to earlier cross-agency tables that had already set a collaborative tone, “the social framework, leadership table…was really…instrumental to sort of set the tone,” and to ongoing mechanisms (e.g., action tables) that keep partners in conversation, stressing that “our pie has to be connected…part of the same pie.” Moreover, these organizational connections and processes were anchored in real, reachable people, which fostered communication routines. From a school-district vantage point, Taylor praised “people who are reaching out and kind of keeping people connected and asking good questions” at the community level. Participants also described cyclical routines that kept collaboration moving without overburdening partners. From a school-division perspective, Kim captured the everyday effect of these scaffolds: “the protocol gives us a reason to connect and talk,” which in turn seeds further joint planning and support for families. Anchored in joint vision, structure functions less as bureaucracy than as a humanized ritual of return that kept trust warm; communication processes were cyclically facilitated by reachable contacts and ongoing meetings.

#### 5.2.2 Transparency and uneven engagement

At the same time, participants described how communication structures were unevenly inhabited. Rowan noted uneven participation, with a small core often carrying the work. Jordan stressed transparency requires full participation, which wasn’t always present. For several, FOIP became a flashpoint. Casey reflected that “people used FOIP as a reason not to share, when in fact FOIP allowed for more sharing than we realized.” Avery noted, “there’s a lot of legislation, there’s a lot of legal challenges and obstacles that would inhibit one from being able to share information freely amongst those that are involved in the protocol. … I think it took two solid years…for the protocol to come to fruition from start to finish.” Riley emphasized that several FOIP issues “depend[ed] on which organization you deal[t] with.” Morgan gave sharper voice to these frustrations, describing how their organization was sometimes left out of ongoing communication. Others echoed that schools, in particular, were inconsistent in sharing VTRA information or delayed in relaying critical updates, creating confusion and frustration across agencies. In addition, participants voiced uncertainty about who was responsible for follow-up actions, an ambiguity that could cause delays or duplication. Together, these gaps suggested that the limits of communication were not only about willingness or privacy law but also about uneven organizational practices that left cracks in the collaborative system.

#### 5.2.3 Managing role changes

Participants also reflected on the disruption caused by inevitable turnover and shifting roles. Casey described the difficulty of “bringing new people up to speed when you have already built momentum.” Kim, recalling the frustration of “re-explaining the whole history every time someone new joined.” Similarly, Riley admitted that “sometimes it felt like you were starting over every 6 months.” Despite supportive structures, staffing changes destabilized and slowed system capacity, amplifying the burden on those left to continually rebuild and sustain institutional memory.

#### 5.2.4 Bringing communication into view

Taken together, these accounts show that communication structures were both enabling and precarious. They allowed information to flow and built rhythms of return that supported trust. Yet they also revealed fragility when transparency faltered, engagement waned, or roles shifted too often to sustain continuity. These participants’ voices bring into view that collaboration depended less on perfect processes than on the continual renewal of communicative ties, holding partners at the table, rebuilding trust after disruptions, and keeping information in motion even when systems hesitated. At the same time, participants acknowledged that uneven follow-up, confusion about responsibilities, and FOIP misunderstandings could quickly unravel momentum, reminding us that communication is a site of risk as well as possibility.

### 5.3 Theme 3: cross-sectoral relationships and joint purpose

When participants reflected on what sustained the VTSP protocol, they often reached beneath processes and leadership to name something more elemental: the relationships and sense of joint purpose that predated and undergirded the work. The protocol, they suggested, did not generate trust on its own; rather, it sat atop a fabric of relational ties woven through years of working together across schools, policing, health, and community services. These ties created a substratum of familiarity and confidence that allowed the protocol to take root. Alongside these relationships was a shared moral horizon, a collective commitment to protecting children and youth, that provided direction even when systems faltered or meetings grew difficult. Trust and purpose worked together: the relational soil held people at the table, and the shared purpose oriented their efforts toward something larger than institutional interests. What emerges here is less a story of structures or training than of the invisible groundwork, the already-existing trust and shared sense of why the work mattered, that scaffolded and sustained collaboration.

#### 5.3.1 Relational trust as substratum

Again and again, participants emphasized that the VTSP protocol worked because it rested on pre-existing cross-sectoral relationships that began with the social framework. Madison indicated that the protocol sat on trust that was already there, noting that the protocol would not have taken hold if the partners had been strangers to each other. Kim emphasized the closeness of operational partnerships with the County and the RCMP, forged through frequent collaboration. For both, the visible procedures of the protocol gained their strength from invisible ties of familiarity and respect. This trust also derived from knowing the person on the other end of the phone, as Casey recalled, a conviction that actions will align with verbal commitments. Avery spoke to the emotional dimension of this substratum, noting that partners could walk into a tense meeting and remain at the table because they knew each other as people, not just as roles. Taylor reinforced this, reminding that trust also meant not overlooking grassroots actors: “Do not forget about the grassroots people who actually know your legs of your community members… make sure you included your grassroots people that are doing the legwork.” Taylor’s caution highlights that sustaining trust was not only about senior relationships but also about valuing frontline workers who carried community knowledge. These reflections underscore how trust was not only professional but personal, a sense of being recognized as whole people that softened the edges of tense encounters and kept collaboration possible.

Several participants made clear that this relational substratum long predated the VTSP itself. Avery described the protocol as “almost a baby that came from the social framework,” while Madison was even more direct: “I think that it has very little, actually, to do with VTSP… I do not think that it’s because of this protocol or because they have signed on that they stay involved and invested. I think that it is the bigger picture.” Moreover, Alex acknowledged that community resources help achieve their broader purpose of proactively supporting mental health. For these participants, the protocol was not the source of collaboration but an extension of a deeper community ethos.

Yet this trust was not evenly distributed. Rowan contrasted contexts, noting that in some settings a privacy-guarded culture was “a little bit adverse to sharing,” which made collaboration harder without support and training. When relationships were underdeveloped, Alex characterized online meetings with unfamiliar representatives as “awkward,” challenging productive dialogue. Across the school–RCMP interface, by contrast, repeated interactions in stable roles did much of the trust-building. Riley, speaking from the policing side, offered a concrete contrast: “I have a really good working relationship with my schools… they know me, and they trust me… we share information openly, but… for a regular member… it’s much more difficult for them to get it, than for me.” Kim, from the education side, added that RCMP remained among their closest operational partners “because we work with them so much.” These accounts show how proximity and role-stability, more than policy alone, sustain rapport with key contacts and keep information moving when it matters. Where contact was frequent and roles were stable, the protocol’s scaffolds had something to grip; where ties were thin, the same scaffolds felt brittle. The unevenness revealed that trust was less a universal feature of the protocol than a fragile inheritance of past work together, which determined whether collaboration flowed or was frangible. The strength of collaboration, then, was deeply contingent, less on the protocol itself than on the social fabric into which it was woven.

#### 5.3.2 Joint purpose as sustaining ethos

Alongside trust, participants spoke of a shared sense of purpose that kept them at the table even when the work was difficult. Rowan called the early cross-sector groundwork “an important primer,” for later collaboration. Many participants identified the pivotal role of an orienting shared framework that was collaboratively devised through a committee of organizational stakeholders. With the addition of the protocol and the tone of collaboration set, Avery highlighted the synergistic momentum built: “We knew, everybody was rowing in the same direction.” This synchronicity allowed them to navigate the inward and interorganizational undulations they encountered when executing joint work. Madison likewise stressed the need for a “bigger picture understanding of systems of community safety and wellbeing,” situating daily tasks within a shared moral horizon. When purpose risked being eclipsed by procedure, Jordan cautioned against “put[ting] process above safety,” arguing that information must be shared when safety is at stake. This shared ethos often acted as a corrective when processes faltered. Riley grounded the purpose: “our main concerns are youth and children and providing the services we can for them to succeed.” Especially when coordination was hard, Rowan described returning to a common horizon, “what brings us together? What is the universal thing that we are all trying to do?” These reflections show how a shared moral compass, centered on safety, wellbeing, and the needs of young people, oriented the work beyond organizational silos and helped partners persist through difficult moments.

#### 5.3.3 Bringing relational groundwork into view

Collaboration in these stories did not arise solely from the VTSP protocol, but from the continual renewal of the network’s relational and moral groundwork. Trust, cultivated over years of cross-sectoral collaboration, acted as the soil that allowed structures to take hold, while a shared purpose, the safety and wellbeing of children, served as the horizon that oriented action. However, trust was uneven, inherited from past encounters. Participants noted that when relational inheritance was thin or purpose wavered, collaboration could quickly sag, exposing vulnerabilities that mirrored those in other themes: reliance on certain people, drift in engagement, and fragility when institutional memory was lost. Although fragile, participants described how these foundations steadied the work when systems hesitated and meetings grew heavy: trust allowed partners to remain at the table, and shared purpose reminded them why it mattered to keep returning. Thus, participants framed the VTSP less as the cause of collaboration than as one expression of a broader culture of co-action and commitment, which partners leaned on when procedures or participation strained.

### 5.4 Theme 4: capacity building and shared language

When participants described how collaboration endured across years of change, they repeatedly returned to the work of building capacity. Training, re-education, and common language provided the scaffolding that allowed diverse organizations to keep moving together despite turnover, shifting roles, and uneven histories of engagement. Through ongoing learning and the cultivation of a shared vocabulary, especially around trauma-informed practice, participants found ways to bridge their institutional differences and sustain collective momentum. What emerges here is not alignment as an abstract state but as something actively sustained through training, re-education, and the disciplined work of speaking the same language across organizations.

#### 5.4.1 Training and re-education across turnover

Several participants emphasized that the continuity of the protocol depended on training that could outlast the churn of staffing changes. Casey described how her division has built training into routine practice, so knowledge survives personnel changes: “There’s always somebody who understands its effectiveness… we have built it into our practice in terms of these are necessary trainings to have certain positions in our organization.” She also pointed to ongoing training for administrators and school-based leads who know the steps and who to call during activations. In effect, training is converted from a one-off orientation to a standing practice that moves knowledge from people to roles; it renders activation more predictable and lowers the risk that memory gaps stall a response. This routinization also resonates with the wider “cadence” described by participants; partners expect to revisit shared material, which helps keep the work alive between incidents.

Pat, by contrast, flagged a gap in formal onboarding across leadership transitions: “I’m going to go to the new officer in charge, and I’m going to explain to them about VTSP and what it is and why it’s there… That’s as much onboarding as you are going to get… No, I do not think there’s formal, you know, skilling up, if you will, to have people ready to be able to implement this.” Where onboarding leans on personal hand-offs rather than structured skilling, knowledge remains unevenly distributed and contingent on who is present. The result is procedural variability across leadership changes, precisely the kind of fragility participants elsewhere linked to over-reliance on key individuals. He also noted turnover-related knowledge loss: “There are a few names that are no longer here… and I’m not sure, with the exception of maybe one, that the knowledge has transferred to their successor,” calling this “a real weakness of the model… that it’s not as transferable as it could be.” This points to knowledge that is “sticky” to persons rather than codified in the system; each departure exposes seams in collective memory and requires those who remain to re-teach the work. Participants’ accounts suggest that transferability is not only a technical problem (documents, checklists) but a relational one; new leaders must be brought into the shared ethos as well as the steps.

From the policing side, Riley underscored the value of refreshers: after initial VTRA I/II, he has “taken it again in the last couple years… just to get retrained or just refreshed is huge,” agreeing with the idea of redoing training every couple of years to keep skills current. Refreshers do more than update procedures; they renew a common vocabulary (e.g., VTRA/TES language) and resynchronize partners’ expectations, which helps absorb turnover without losing coherence. In this sense, recurrent training functions as institutional memory in motion, carrying shared understandings forward even as people change.

#### 5.4.2 Common language and trauma-informed approaches

Alongside training, participants spoke of the power of cultivating a shared vocabulary that crossed organizational borders. Madison emphasized that TES training helped partners “identify how systems are connected and how influential they are with one another,” adding that it “shed light on maybe how we work, or need to work with some systems,” and that leaders “might have to approach things differently,” which “was… really, really important and influential.” Taylor extended this, pointing to the common language of VTRA and TES training as a critical glue that guided collective work: “the training from TES … and Kevin Cameron… it’s a shared language. It’s a shared approach.” Within the protocol, Madison clarified that a collective understanding emerged of “how systems are connected and how influential they are with one another.” She attributed this system lens to a deepened understanding of collaboration and how to engage with diverse stakeholders: “I think the TES training itself sort of shed light on maybe how we work, or need to work with some systems. And we have been able to name that in a different way, which just allows you to realize that you might have to approach things differently. so that the training was. really, really important and influential.” At the same time, Taylor cautioned that gaps emerged when not all responders had been trained, leaving uneven capacity across organizations. These insights suggest that language was not a neutral medium, but a unifying scaffold. Shared language did not just produce coherence; it set a standard for care, aligning internal responses and the public voice to avoid re-traumatizing audience.

Participants also located this shared language inside a trauma-informed lens. Rae described the practical shift this lens creates: attending both to “how people showing up in our systems may… have some of these traumas,” and to “the event itself and the impacts, and then those ripples and the multiple impacts… and how do we plan accordingly?” Across interviews, leaders used the TES phrase “lowering the anxiety of the system” as a touchstone. Madison noted that this focus “has changed how decisions have been made,” and Casey said, “we use the principles from traumatic event systems… bringing down the anxiety of the system… far beyond the specific events that it’s relevant to… in our everyday leadership.” In practice, this shared shorthand gave multi-sector teams a way to name the same problem and move together in tense moments.

Finally, participants linked a common language to outward-facing responsibility. Kim explained that, especially in significant situations, the division supports schools “with communication out to families… to make sure that the communication is clear,” illustrating how a shared frame shapes both internal coordination and public messaging. Participants emphasized a trauma-informed stance in community communications: using plain, non-alarmist language, pacing what is shared; naming what is known and unknown; avoiding unnecessary detail; identifying a trusted central voice; and pointing families to supports. In this way, shared language worked not only to keep partners coherent but to meet the community carefully and consistently, minimizing re-traumatization while sustaining trust.

#### 5.4.3 Bringing capacity and shared language into view

Beyond initial enthusiasm, these accounts show that sustaining cross-sectoral collaboration required continual work to build system-wide capacity through re-training and cultivating a common language that could facilitate dialogue among diverse actors. Participants made it clear that collaboration was not a given, but rather something fragile, always at risk of sagging under the weight of staff changes, institutional silos, or competing vocabularies. Training and re-education served as the practice of carrying institutional memory, while trauma-informed approaches and shared vocabulary created coherence across differences. Participants described the shared language, developed through VTRA and TES training, as a unifying scaffold that transcended organizational boundaries. The common vocabulary provided a systemic map of how organizations connect and influence one another, enabled predictable coordination, and supported trauma-attuned judgment when procedures lagged. It also set a standard for public communication, characterized by plain language, a paced tone, and a non-alarmist approach, minimizing re-traumatization and sustaining community trust. Collaboration, in these stories, endured not because organizations were naturally aligned but because participants engaged in the slow, repeated labour of building and renewing the very capacities that made alignment possible.

### 5.5 Fragility as a cross-cutting thread

Across the four themes, collaboration was described as both resilient and precarious. Leadership, communication, relationships, and capacity each sustained the work, but each also carried vulnerabilities—overreliance on individuals, misunderstandings of FOIP, erosion of trust, or the churn of turnover. Participants also cautioned that fragility could emerge when grassroots actors were overlooked or when processes became too rigid, reminding us that even stabilizing structures could themselves create new risks. What participants brought into view was collaboration not as a stable state but as a fragile accomplishment, enduring only through continual reinvestment. This fragility was not always explicitly stated but was evident in the disruptions and cautions expressed across interviews. This paradox, in which collaboration is both strong and fragile, gave the work its urgency and made renewal, rather than stability, the central practice for sustaining the protocol. Participants’ remedies were equally concrete—rotate backbone functions, refresh training, clarify FOIP-compliant pathways, and keep a live roster of reachable contacts—so that renewal is structured, not *ad hoc*.

## 6 Discussion

This study examined how stakeholders and organizations within Strathcona County’s Violence, Trauma, and Suicide Prevention Protocol collaborate and exchange information to address the mental health, safety, and wellbeing of children and youth. Across the interviews, collaboration emerged as a fragile accomplishment: strong enough to sustain coordinated action across education, health, policing, and community services, yet precarious enough to require continual renewal through leadership, communication, relationships, and capacity building. This finding is significant because it extends existing scholarship on interagency collaboration and collective impact by showing not only what enables multi-sector collaboration to begin, but what holds it together over time.

The findings respond directly to the two research questions guiding this study. First, stakeholders collaborate and exchange information through a combination of formal structures and relational practices. The VTSP Protocol, VTRA and TES processes, stewardship meetings, community leads, shared forms, and recurring touchpoints provide the procedural scaffolding through which information can move across organizational boundaries. However, participants made clear that these structures are insufficient on their own. Information exchange depends on trust, role clarity, shared language, and the presence of people who know whom to call, how to interpret privacy obligations, and how to keep partners engaged when uncertainty or crisis emerges. Second, the success of the VTSP Protocol rests on underlying strategies and relational dynamics that are both structural and affective: distributed leadership, backbone coordination, recurring training, relational trust, shared purpose, trauma-informed communication, and an ongoing commitment to keeping children and youth at the centre of the work. These findings suggest that coordinated community responses are not simply implemented as stable institutional arrangements; they are continually reconstituted through everyday relational, communicative, and capacity-building labour. Relational Coordination Theory provides the overarching interpretive lens for the discussion that follows, while related scholarship is used to situate and extend the findings.

### 6.1 Collaboration as continual renewal rather than stable structure

A central contribution of this study is its depiction of collaboration as continual renewal. Viewed through Relational Coordination Theory, continual renewal reflects the ongoing relational and communicative work required to sustain coordination across organizational boundaries. Much of the collective impact literature identifies the formal conditions that support cross-sector collaboration, including a common agenda, mutually reinforcing activities, continuous communication, shared measurement, and backbone support (12). These conditions were clearly visible in the VTSP Protocol. Participants described a shared moral agenda centered on child and youth safety, shared methodology through VTRA and TES, continuous communication through committees and meetings, and a backbone role played largely by Family and Community Services and key community leads.

Given this need for ongoing renewal, the findings also complicate collective impact theory literature by positioning these conditions as dynamic design features. The common agenda must be reanimated when partners drift toward organizational priorities. Continuous communication must be protected against turnover, uneven participation, and misunderstandings about information sharing. Backbone support must be resourced and distributed so that it does not become overly dependent on a single person or agency. Implementation science frameworks frequently highlight the fluid role of context in iteratively shaping the success of initiatives, including intersecting organizational factors (e.g., leadership, culture, resources) (45). In this sense, the VTSP Protocol illustrates collective impact less as a model that is established once and then sustained by design, and more as a living system that depends on ongoing recommitment.

This finding aligns with more recent critiques and extensions of collective impact theory literature. Kania et al. (13) argue that collective impact efforts must be understood as adaptive networks of learning, alignment, and integration rather than technical coordination mechanisms alone. Similarly, Wolff et al. (14) caution that collective impact initiatives can become overly managerial if they fail to attend to power, participation, and community ownership. Participants in this study similarly emphasized that the protocol’s strength did not lie only in formal agreement but in the relational and ethical commitments that animated it. The work endured because partners repeatedly returned to the question of what they were trying to do together: protect and support children and youth before crises escalated.

### 6.2 Leadership as relational infrastructure

The finding that leadership functioned as relational infrastructure is best understood through Relational Coordination Theory, which emphasizes the role of shared goals, shared knowledge, mutual respect, and frequent, timely, accurate, and problem-solving communication in coordinating interdependent work (18, 19). The VTSP findings highlight the perceived leadership practices of key connectors and brokers identified through the study’s social network analysis. Notably, participants rarely described leadership in hierarchical terms. At times catalytic and visionary, they spoke of leadership as convening, brokering, holding, bridging, pacing, and keeping people connected. These practices helped sustain the relational and communicative conditions through which diverse cross-sectoral partners could act together across organizational boundaries. In synergistic interagency partnerships, Wiggins et al. (35) found that leadership was “dynamic, empowering, and enthusiastic” (p. 98); leaders were described as capable managers (i.e., coordination, responsibility), but also magnetic culture builders who inspired collective engagement and candor. Viewed ecologically, leadership mattered to the protocol because youth mental health, trauma response, violence prevention, and suicide prevention are shaped across multiple interacting systems; no single organization can interpret or respond to the whole situation alone (16, 17). Leadership, therefore, operated not simply as administrative coordination but as a relational mechanism for holding an ecological community response together.

This interpretation also clarifies the relationship between the findings and the broader collective impact literature. The VTSP Protocol included recognizable collective impact conditions, including shared purpose, continuous communication, mutually reinforcing activity, and backbone support (12). However, participants’ accounts suggest that these conditions were not stable design features that, once established, sustained themselves. Rather, they had to be continually renewed through relational labour, communication, trust-building, and problem solving. This finding is consistent with more recent collective impact scholarship that frames cross-sector collaboration as adaptive, relational, and dependent on ongoing learning and alignment rather than technical coordination alone (13, 14). In the VTSP Protocol, broker leadership made collaboration possible, but it also exposed the system to succession risk when too much of the backbone function became concentrated in key individuals or one coordinating agency. Network scholarship suggests that brokers who connect otherwise separate actors or clusters can provide essential reach and integration, but they may also create vulnerability when too much network coherence depends on a small number of people (40). The implication is that leadership must be intentionally distributed, documented, and renewed so that the relational conditions of coordination can persist when specific people or roles change (18, 19).

### 6.3 Communication, information sharing, and the limits of formal process

The second major finding concerns communication as both enabling and precarious. Participants repeatedly emphasized that shared processes, including VTRA, TES, stewardship meetings, common forms, and designated leads, helped create predictable pathways for information exchange. This finding speaks directly to Relational Coordination Theory’s emphasis on frequent, timely, accurate, and problem-solving communication. This finding is consistent with interagency collaboration literature, which identifies communication quality and frequency as key facilitators of effective collaboration (30, 35). The VTSP Protocol provided a reason for partners to gather, a common language for interpreting concern, and a structure for coordinated action.

However, the findings also show that formal processes do not eliminate communicative fragility. Participants described uneven participation, uncertainty about follow-up responsibilities, differences in organizational practice, and persistent confusion about privacy legislation. Indeed, research has frequently found that matters of confidentiality (e.g., privacy laws, consent processes) are perceived as hindrances to inter-organizational information sharing (30, 46–48). As a point of emphasis, our participants also described privacy concerns as a barrier to information sharing, while others suggested that FOIP was sometimes misunderstood as preventing communication when it may have permitted more sharing than partners realized. This finding matters because it suggests that information-sharing barriers are not only legislative or technical; they are also interpretive and relational. Partners need shared legal understanding, but they also need trust, confidence, and role clarity to act on that understanding.

This finding extends existing literature on interagency collaboration by showing how communication barriers operate in the lived experience of practitioners. Previous reviews identify communication and information sharing as both major facilitators and major hindrances in collaborative work (30, 35). The VTSP findings add nuance by showing that communication structures must be continually inhabited by people who understand the protocol, know one another, and are confident in their responsibilities. Without these human and relational conditions, structures can become hollow, existing on paper without reliably supporting coordinated action.

### 6.4 Relational trust and shared purpose as the ground of collaboration

Participants consistently described relational trust as the substratum of the VTSP Protocol. The protocol did not create collaboration from nothing; rather, it rested on a longer history of community relationships, including prior cross-sector work through the social framework. This finding is consistent with interagency collaboration literature, which identifies trust, shared purpose, and networking opportunities as recurring facilitators of collaboration (30, 34, 35). However, the study adds an important temporal dimension: trust was not generated at the moment of activation. It was accumulated through years of prior contact, repeated interaction, and familiarity across organizational boundaries. From a relational coordination perspective, these accounts illuminate the importance of shared goals and mutual respect as foundations for coordinated action.

This finding also resonates with social network scholarship. From a network perspective, collaboration depends not only on the existence of formal ties but on the quality, strength, and distribution of those ties. In connection, research has explored co-located interagency partnerships (22), leveraging school-based coaches or consultants to iteratively cultivate positive relationships with education workers through meaningful interactions that target systemic capacity building (49–52). In our study, participants described stronger information flow where relationships were stable and repeated, such as between schools and the RCMP school resource officers. They also described weaker collaboration where partners were unfamiliar, roles shifted frequently, or meetings occurred online with representatives who did not yet know one another. These accounts demonstrate that the strength of a coordinated response depends on the relational density of the network before a crisis occurs.

A shared purpose was equally important. Stakeholders in high-functioning interagency partnerships reported robust levels of commitment and coordination, supported by strategies that increase distributed leadership and input among partners, including cohering around a common vision (35). Participants repeatedly returned to the safety and wellbeing of children and youth as the moral horizon that allowed partners to move beyond institutional silos. Because these issues exceed the mandate of any one organization, collaboration requires more than coordination; it requires a shared understanding that no single agency can hold the whole problem alone. This finding connects to public health and ecological systems literature, which frames youth mental health and violence prevention as complex issues shaped by interacting individual, family, school, community, and societal factors (17, 20, 22, 24). Shared goals can foster stakeholder engagement (53), provide clear targets for group accountability (54), and allow for mutual benefits (55, 56). The VTSP Protocol worked because partners recognized their interdependence and understood child and youth safety as a shared responsibility.

### 6.5 Capacity building, shared language, and trauma-informed practice

The fourth finding concerns capacity building and shared language as mechanisms for sustaining alignment. Within Relational Coordination Theory, this finding is particularly relevant to the development and maintenance of shared knowledge across organizational boundaries. Participants described training and re-education not as peripheral activities but as essential infrastructure. Prior research has identified professional differences among sectors (e.g., education, healthcare) and disciplines as collaborative barriers (47, 57). In the VTSP protocol, VTRA and TES training provided a shared vocabulary, a common approach to risk and response, and a way of understanding how systems influence one another. This shared language allowed professionals from different sectors to interpret situations more coherently and coordinate action more predictably.

This finding aligns the interagency collaboration and trauma-informed care literatures. For instance, shared training and networking opportunities were identified as importance facilitators of interagency collaboration (30, 34). With a focus on child welfare systems, training was also the most frequently reported implementation strategy identified in a rapid review the evidence on the adoption of trauma-informed care (TIC) practices (58). As opposed to a one-time capacity-building event, Bunting et al.’s (58) review also found that initiatives to systemically implement TIC practices placed an emphasis on iterative support strategies; that is, staff support was continually embedded through approaches such as learning collaboratives, ongoing staff training, or developmental models (e.g., coaching, mentoring). Similarly, our participants emphasized the importance of refreshers, onboarding, and role-based training so that knowledge remains attached to positions rather than only to individuals. Without this renewal, the VTSP protocol becomes vulnerable to knowledge loss, especially in systems marked by staff turnover, changing leadership, and uneven organizational memory.

The shared language of TES also supported trauma-informed practice. Participants described the importance of lowering the anxiety of the system, using plain and non-alarmist language, pacing communication, naming what is known and unknown, and avoiding unnecessary detail that could re-traumatize families or communities. With threat assessment and trauma-informed care implementation, some research has found that organizational training is associated with gains in related knowledge, attitudes, skills, and/or confidence levels (58, 59). Building systemic capacity in TIC and threat assessment also aligns with public health approaches that emphasize prevention, protective environments, relational connection, and coordinated intervention to lessen harm (20). It also shows that communication is not merely informational. It is ethical and affective. How partners communicate during moments of high intensity helps shape whether the system escalates anxiety or contains it.

### 6.6 Implications for coordinated community responses

The findings have several implications for communities and school authorities seeking to develop similar coordinated responses. First, communities should build relational infrastructure before it is needed. Trust cannot be improvised during crisis. It must be cultivated through regular cross-sector meetings, shared training, and opportunities for partners to know one another beyond formal roles.

Second, backbone functions should be distributed rather than concentrated in a single champion. The VTSP Protocol demonstrates the importance of convening, brokering, documenting, and sustaining communication, but it also shows the risks of over-reliance on particular individuals. Communities should codify backbone functions through role descriptions, shared templates, rotating leads, documented hand-offs, and live contact lists.

Third, privacy and information-sharing protocols require more than legal compliance. Partners need shared interpretation, scenario-based training, and practical tools that help them understand what can be shared, with whom, under what conditions, and for what purpose. Clarifying these pathways may reduce hesitation and prevent safety-related information from stalling at organizational boundaries.

Fourth, recurring training is essential to sustainability. VTRA, TES, trauma-informed communication, and onboarding should be built into organizational routines rather than offered only at implementation. This is especially important in systems where turnover is expected. Training functions as institutional memory and helps maintain shared language across sectors.

Finally, coordinated community responses should attend more deliberately to whose voices are present and absent. While the VTSP Protocol demonstrates strong cross-sector professional collaboration, the broader collective impact and interagency collaboration literature increasingly emphasizes equity, participation, and community ownership (13, 14, 60–63). Future development of the model could consider how youth, families, and community members might contribute safely and meaningfully to protocol design, review, and renewal.

### 6.7 Research contributions to interagency collaboration

Taken together, these findings make several contributions to the threat assessment and interagency collaboration literature. In a systematic review of threat assessment research related to K-12 schools, Ross et al. (59) found that 18 of 23 articles focused on some iteration of either the Virgina Student Threat Assessment Guidelines (VSTAG) or Comprehensive School Threat Assessment Guidelines (CSTAG). A dearth of research has studied the implementation and impacts of the VTRA model (31, 59). Moreover, Jackson and Viljoen (31) conducted a review of five prevalent school threat assessment models, finding that most approaches centred on school-level teams. In contrast, they found that VTRA was one of two models that incorporated community-level collaborators within the threat assessment process. Thus, our findings contribute to what is known about the implementation of interagency collaboration within the VTRA model, demonstrating what a coordinated community response can look like in practice.

## 7 Conclusion

This study examined how stakeholders within Strathcona County’s Violence, Trauma, and Suicide Prevention Protocol collaborated across education, health, policing, municipal, and community sectors to support the mental health, safety, and wellbeing of children and youth. The findings show that the VTSP Protocol is not sustained by formal agreement or procedural design alone. Rather, it depends on the continual renewal of relational, communicative, and capacity-building practices that allow diverse partners to act together across organizational boundaries.

This study contributes to understanding coordinated community responses by showing that collaboration is both powerful and precarious. The participants in this study described leadership as a relational infrastructure, communication structures as necessary but uneven, cross-sectoral relationships and joint purpose as the ground of collaboration, and capacity building and shared language as essential to sustaining alignment. Across these themes, collaboration emerged as a fragile accomplishment: strong enough to support coordinated action, yet vulnerable to turnover, uneven participation, unclear follow-up, privacy misunderstandings, and over-reliance on key individuals.

Theoretically, the findings affirm the value of understanding youth mental health and community safety through an ecological lens, while also demonstrating the importance of relational coordination in sustaining cross-sector work. This study highlighted that no single organization can hold the full picture of a young person’s needs or risks. What is needed is a coordinated response with shared goals, shared knowledge, mutual respect, and communication that is timely, accurate, and oriented toward problem-solving.

For communities and school authorities seeking to develop similar protocols, the implications are practical. A coordinated community response must be intentionally resourced, regularly renewed, and distributed across roles rather than concentrated in a small number of champions. Training, shared language, clear information-sharing pathways, current contact structures, and trauma-informed communication are essential; they are core conditions for sustainability.

In a context of rising concern about youth mental health, suicide, trauma, and community safety, the VTSP Protocol offers an important example of what it takes to hold systems together around children and youth. Its lesson is not simply that collaboration matters, but that collaboration must be actively sustained. Protocols may create the conditions for coordination, but it is the ongoing human work of trust, communication, shared purpose, and collective responsibility that keeps a coordinated community response alive.

## Data Availability

The datasets presented in this article are not readily available because data not available due to ethical restrictions. Requests to access the datasets should be directed to Sharon Friesen, sfriesen@ucalgary.ca.
