# Supplementary material for: Holding collaboration together: a community response using a violence threat and suicide prevention protocol
Source: Front Public Health. 2026 Sep 16;14:1933435. doi: 10.3389/fpubh.2026.1933435 (PMC13623696; doi:10.3389/fpubh.2026.1933435)
Supplement: Supplementary file 1 [file Supplementary_File_1.docx]

Supplementary Appendix 1

# 1 VTSP Protocol: Semi-Structured Interview Instrument

The following interview guide is organized into three sections. As the interviews will be semi-structured, it is possible that participants will not be asked some questions. Specifically, if a topic is covered through general conversation during an interview, the associated question(s) will not be asked. Before starting each interview, the following points will be addressed:

- Explain the aim of the research project.
- Briefly describe how the interview will contribute to the research.
- Confirm that participation is completely voluntary.
- Reiterate that they can withdraw at any time without penalty.
- Assure the confidentiality of the responses.
- Request permission to record the interview.
- Explain how the recording will be used and stored.

# 2 Section One - Introduction and Role in VTSP Protocol

- Could you start by telling me about your current role and your professional background?
- How did you become involved with the VTSP Protocol?
- Can you describe your day-to-day responsibilities related to the VTSP Protocol?
- In what ways has your role influenced or shaped the implementation of the VTSP Protocol?

# 3 Section 2 - Reflections on Social Network Analysis Findings

Note. Before asking the Section 2 questions, we will share a high-level overview of the SNA findings with participants. We will discuss general patterns of social interaction uncovered in the VTSP Protocol network.

- After reviewing the summary of our SNA findings, what is your initial reaction? Are there any aspects that particularly resonate with or surprise you?
- Based on the SNA findings presented, how do you perceive the role of collaboration and communication in the success of the VTSP Protocol?
- The SNA highlighted specific patterns of trust and organizational learning within the VTSP Protocol. How do these findings align with your personal experiences?
- Considering the leadership styles identified in the SNA, how do you think leadership has influenced the dynamics and outcomes of the VTSP Protocol?

# 4 Section 3 - Emergent Questions and Partner-Generated Questions

- How has the Traumatic Event System (TES) Model impacted you or your work?
- Reflecting on your experience with the VTSP Protocol, was there a moment when you felt confident it would be successful? What happened that led to this realization?
- Despite changes in leadership and shifts among core team members, the VTSP Protocol has continued to thrive. Can you share how these transitions were managed and what factors contributed to the sustainability of the efforts?
- With the VTSP Protocol including various organizations and initiatives, how have you personally navigated and understood the complexities involved?
